# Non-recovery of renal function is a strong independent risk factor associated with mortality in AKI patients

**DOI:** 10.1590/2175-8239-JBN-2019-0187

**Published:** 2020-07-27

**Authors:** Reginaldo Passoni dos Santos, Ariana Rodrigues da Silva Carvalho, Luis Alberto Batista Peres, Vinicius Daher Alvares Delfino, Cintia Magalhães Carvalho Grion

**Affiliations:** 1 Universidade Estadual do Oeste do Paraná, Programa de Pós-Graduação em Biociências e Saúde, Cascavel, PR, Brasil.; 2 Universidade Estadual do Oeste do Paraná, Centro de Ciências Biológicas e da Saúde, Colegiado de Enfermagem, Cascavel, PR, Brasil.; 3 Universidade Estadual do Oeste do Paraná, Departamento de Medicina, Disciplina de Nefrologia, Cascavel, PR, Brasil.; 4 Universidade Estadual de Londrina, Departamento de Medicina Interna, Seção de Nefrologia, Londrina, PR, Brasil.; 5 Universidade Estadual de Londrina, Departamento de Medicina, Disciplina de Medicina Intensiva, Londrina, PR, Brasil.

**Keywords:** Acute Kidney Injury, Mortality, Risk Factors, Epidemiology, Intensive Care Units, Lesão Renal Aguda, Mortalidade, Fatores de Risco, Epidemiologia, Unidades de Terapia Intensiva

## Abstract

**Introduction:**

Acute kidney injury (AKI) is a recurrent complication in the intensive care unit (ICU) and is associated with negative outcomes.

**Objective:**

To investigate factors associated with mortality in critically ill AKI patients in a South Brazilian ICU.

**Methods:**

The study was observational retrospective involving AKI patients admitted to the ICU between January 2011 and December 2016 of at least 18 years old upon admission and who remained in the ICU at least 48 hours. Comparisons between selected characteristics of survivor and non-survivor groups were done using univariate analysis; multivariate logistic regression was applied to determine factors associated with patient mortality.

**Results:**

Of 838 eligible patients, 613 participated in the study. Men represented the majority (61.2%) of the patients, the median age was 53 years, and the global mortality rate was 39.6% (n= 243). Non-recovery of renal function after AKI (OR= 92.7 [38.43 - 223.62]; p <0.001), major surgery-associated AKI diagnosis (OR= 16.22 [3.49 - 75.38]; p <0.001), and the use of vasoactive drugs during the ICU stay (OR = 11.49 [2.46 - 53.70]; p <0.002) were the main factors independently associated with patient mortality.

**Conclusion:**

The mortality rate observed in this study was similar to that verified in other centers. Non-recovery of renal function was the variable most strongly associated with patient mortality, suggesting that the prevention of factors that aggravate or maintain the AKI episode should be actively identified and mitigated, possibly constituting an important strategy to reduce mortality in AKI patients.

## RESUMO

**Introdução:**

A lesão renal aguda (LRA) é uma complicação recorrente na unidade de terapia intensiva (UTI), e está associada a desfechos desfavoráveis.

**Objetivo:**

Investigar fatores associados à mortalidade em pacientes com LRA, criticamente enfermos em uma UTI do Sul do Brasil.

**Métodos:**

O estudo foi retrospectivo observacional, envolvendo pacientes com LRA internados na UTI entre janeiro de 2011 e dezembro de 2016, com pelo menos 18 anos de idade na admissão e que permaneceram na UTI por pelo menos 48 horas. Comparações entre características selecionadas de grupos sobreviventes e não sobreviventes foram feitas usando análise univariada; regressão logística multivariada foi aplicada para determinar fatores associados à mortalidade dos pacientes.

**Resultados:**

Dos 838 pacientes elegíveis, 613 participaram do estudo. Os homens representaram a maioria (61,2%) dos pacientes, a idade média foi de 53 anos e a taxa de mortalidade global foi de 39,6% (n = 243). Não recuperação da função renal após LRA (OR = 92,7 [38,43 - 223,62]; p <0,001), diagnóstico de LRA associado à cirurgia (OR = 16,22 [3,49 - 75,38]; p <0,001) e uso de drogas vasoativas durante a internação na UTI (OR = 11,49 [2,46 - 53,70]; p <0,002) foram os principais fatores independentemente associados à mortalidade dos pacientes.

**Conclusão:**

A taxa de mortalidade observada neste estudo foi semelhante à verificada em outros centros. A não recuperação da função renal foi a variável mais fortemente associada à mortalidade dos pacientes, sugerindo que a prevenção de fatores que agravam ou mantêm o episódio de LRA deve ser ativamente buscada e incentivada, possivelmente constituindo uma estratégia importante para reduzir a mortalidade em pacientes com LRA.

## Introduction

Acute kidney injury (AKI) is a clinical syndrome, whose main defining characteristic is related to the abrupt and sustained decline of the glomerular filtration rate.^1^^-^ 2 In association with this characteristic, oliguria, water and electrolyte imbalance, and instability of blood pH are also observed.^3^ Also, the complex clinical disorder caused by AKI results in the retention of nitrogenous wastes and increase in extracellular volume.^4^

AKI is a recurrent complication in the intensive care unit (ICU), and is associated with several negative outcomes, including increased length of ICU and hospital stay, greater need for professional care, and a higher mortality rate.^5^^-^ 7 Many aspects influence the variation in the AKI incidence rate, with critically ill patients from developing countries showing worse outcomes.^6^^-^ 8 In a recent international multicenter study, AKI episodes occurred in 44.6% of ICU patients, whereas the incidence of these events was 12.7% in South America.^5^

According to the available literature, in Brazil, the mortality of critically ill AKI patients varies from 15.3% (in the Northeast)^9^ to 53.2% (in the South).^10^ This variation in mortality can be understood in the context of the continental dimensions of Brazil and the great diversity in the allocation of (economic-financial, human, etc.) resources for health care amongst the different Brazilian regions. Therefore, this study aimed to investigate factors associated with mortality in critically ill AKI patients in a South Brazilian ICU.

## Methods

The study report was elaborated following the Strengthening the Reporting of Observational Studies in Epidemiology (STROBE) guidelines.^11^

### Design, Setting, and Patients

We carried out an observational retrospective study in a single ICU in Brazil. The unit is in a university hospital, has fifteen beds, and provides care to clinical and surgical patients of various medical specialties. All patients admitted between January 2011 and December 2016 were included in this research, with ICU-acquired AKI defined and classified according to the Kidney Disease: Improving Global Outcomes (KDIGO)^1^ criteria, who were at least 18 years old upon admission, and remained in the unit at least 48 hours. Patients with AKI acquired outside the ICU, those with serum creatinine >4.0 mg/dL upon admission, and with chronic kidney disease were excluded.

### Variables

Data collection occurred between October 2016 and January 2018, and included variables of clinical-epidemiological features (age; gender; race; comorbidities; diagnosis and clinical conditions at ICU admission (ICU-ad), serum creatine and oliguria at ICU-ad; urine output in the first 24 hours; scores of the second version of the mortality prognostic system Acute Physiology and Chronic Health Evaluation (APACHE II),^12^ and length of ICU and hospital stay).

Furthermore, data were collected on occurrences during the ICU stay, including general occurrences (need for mechanical ventilation, intravenous contrast, blood transfusion, surgeries, infection, and sepsis), biochemical imbalance (hyponatremia - sodium serum concentration <135 mEq/L; hypernatremia - sodium serum concentration >145 mEq/L; hypokalemia - potassium serum concentration <3.5 mEq/L, hyperkalemia - potassium serum concentration >5.5 mEq/L; hypoglycemia - glucose serum concentration <70 mg/dL; hyperglycemia - glucose serum concentration >140 mg/dL; metabolic acidosis - arterial blood pH <7.35 and bicarbonate serum concentration <22 mEq/L), need for drugs (vasoactive drugs, diuretics, nonsteroidal anti-inflammatory drugs, vancomycin, amphotericin B, polymyxin B, and aminoglycosides); and fluid balance (days of oliguria, positive and negative fluid balance, as well as accumulated fluid balance). Oliguria was defined as urine output below 400 mL/day. Fluid balance was considered as the difference between fluid gains and losses by the patient over 24 hours. Thus, we considered that patients with positive fluid balance (or water overload) presented fluid gains greater than losses (in 24 h). Accumulated fluid balance was considered as the result of the patient’s general water balance at the end of the ICU stay.

Organ dysfunction (hematological, cardiovascular, hepatology, respiratory, and multiple) was analyzed considering the current definitions.^13^ Additionally, we collected variables related to the timing of the AKI diagnosis (KDIGO criteria for diagnosis and stage, main associated etiology, and need for renal replacement therapy (RRT)). Finally, we evaluated the recovery of renal function at ICU discharge, classifying the recovery in three levels: 1) full recovery - absence of AKI criteria; 2) partial recovery - drop in the AKI stage; 3) non-recovery - maintenance of the AKI stage.^14^

### Statistical Analysis

We determined the mortality rate by the number of patients with AKI who died in the ICU. The mortality risk factors were determined by performing comparative analysis between groups of survivor and non-survivor patients. Categorical variables are presented as absolute and relative frequency and continuous variables as medians and interquartile range (IQR 25% - 75%). Comparisons between groups were carried out applying Chi-square or Fisher’s exact test and Mann-Whitney’s U-test, for categorical and continuous variables, respectively.

Variables that in the bivariate analysis presented *p*-value <0.20 were selected for the multivariate logistic regression model using the stepwise method. In the final model, variables with variance inflation factor greater than ten were excluded to avoid multicollinearity problems. The Hosmer-Lemeshow test and area under the receiver operating characteristic curve were used to assess the discrimination and calibration of the final model, respectively. Also, we constructed a Kaplan-Meier curve with log-rank test to determine survival rates for the different subgroups of recovery of renal function post-AKI in ICU. We considered *p-*values <0.05 as statistically significant. All analyses were performed in the XLSTAT version 2018.

### Ethical Aspects

The study received approval from the Ethics Committee of the authors’ institution, under approval number 1.622.962. There was no need for informed consent. Nonetheless, we declare that all local and international ethical precepts have been respected, including those contained in the Declaration of Helsinki.

## Results

**Figure 1:**
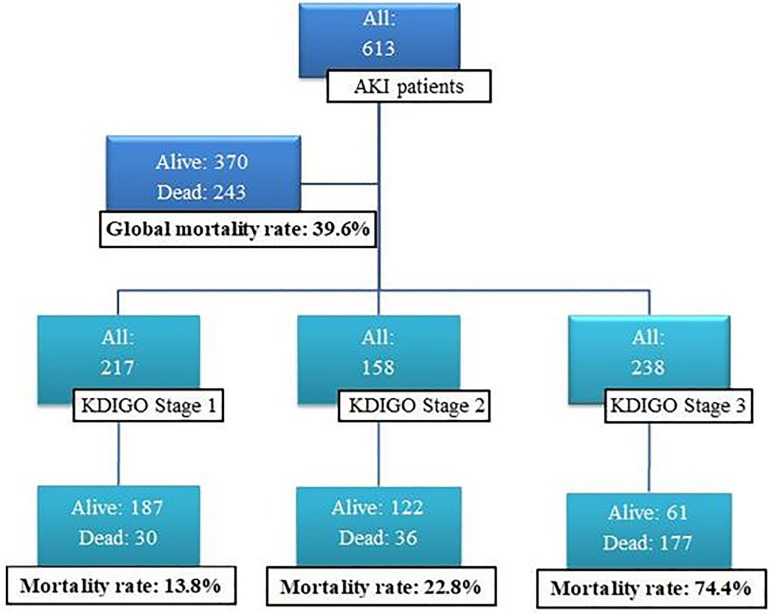
Mortality rate global and according the KDIGO stage. KDIGO - Kidney Disease Improving Global Outcomes.

Of 838 eligible patients, we excluded 64 with AKI acquired before ICU-ad, 52 with serum creatinine >4.0 mg/dL, and 109 with chronic kidney disease. Thus, in this study, 613 AKI patients were included. The median age was 53 (38-67) years, and non-survivors were older. In the mortality rate analysis, we could determine that the global mortality continued at 39.6% (*n=* 243) and that, among those with KDIGO stage 3, the mortality amounted to 74.4% (Fig. 1).

When comparing the data collected in the ICU-ad, there was no difference between the groups for serum creatinine (survivors: 1.1 [0.7-1.7] mg/dL *vs.* nonsurvivors: 1.1 [0.8-1.6] mg/dL; *p-*value 0.547), but oliguria upon admission was more frequent among non-survivors (*n=* 25; 10.3% *vs.* survivors group: *n=* 11; 3.0%; *p-*value <0.001). Similarly, APACHE II score was also significantly higher among those who died (median 28 [23-31] points *vs.* survivors group: median 24 [19-29] points; *p-*value <0.001) (Table 1).

**Table 1:** Clinical-epidemiological features of patients.

Variables	All patients (*n*= 613)	Survivors (*n*= 370)	Non-survivors (*n*= 243)	*p*
Age (years)	53 (38 – 67)	51 (38 – 66)	57 (41 – 69)	**0.044**
Gender (male)	375 (61.2%)	239 (64.6%)	136 (56%)	**0.039**
Race (Caucasian)	525 (85.6%)	317 (85.7%)	208 (85.6%)	0.928
Comorbidities				
Hypertension	250 (40.8%)	154 (41.6%)	96 (39.5%)	0.662
Diabetes	99 (16.2%)	58 (15.7%)	41 (16.9%)	0.778
Cancer	50 (8.2%)	32 (8.6%)	18 (7.4%)	0.690
Diagnosis at ICU-ad				
Surgical	359 (58.6%)	240 (64.9%)	119 (49%)	**< 0.001**
Clinical	254 (41.4%)	130 (35.1%)	124 (51%)	
Clinical conditions at ICU-ad				
Mechanical ventilation	542 (88.4%)	315 (85.1%)	227 (93.4%)	**0.003**
Nosocomial infection	160 (26.1%)	84 (22.7%)	76 (31.3%)	**0.023**
Polytrauma	160 (26.1%)	101 (27.3%)	59 (24.3)	0.460
Creatinine at ICU-ad (mg/dL)	1.1 (0.8 – 1.7)	1.1 (0.7 – 1.7)	1.1 (0.8 – 1.6)	0.547
Oliguria at ICU-ad	36 (5.9%)	11 (3.0%)	25 (10.3%)	**< 0.001**
Urine output				
(first 24 hours, liters)	2.4 (1.5 – 3.4)	2.4 (1.6 – 3.4)	2.3 (1.3 – 3.5)	0.116
APACHE II	26 (21 – 30)	24 (19 – 29)	28 (23 – 31)	**<0.001**
Length of stay (days)				
Hospital	23 (12 – 39)	28 (17 – 43)	15 (9 – 31)	**<0.001**
ICU	12 (6 – 22)	12 (6 – 21)	12 (5,5 – 24)	0.708

ICU-ad: intensive care unit admission; APACHE: Acute Physiology and Chronic Health Evaluation.

During the ICU stay, the need for mechanical ventilation was greater among non-survivors (median 10 [5-20] days *vs.* survivor group: median 9 [4-17] days; *p-*value <0.001), as well as the occurrence of sepsis (*n*= 61, 16.5% vs. survivor group: *n*= 102, 40.2%; *p*-value <0.001) and metabolic acidosis (*n*= 252, 68.1% *vs.* survivor group: *n*= 217, 89.3%; *p*-value <0.001). Non-survivors had less need for NSAIDs (*n*= 98, 40.3% *vs.* survivor group: *n*= 216, 58.4%, *p*-value <0.001), but higher fluid balance alterations and higher frequency of organ dysfunction (Table 2).

**Table 2:** Occurrences during the length of ICU stay.

Variables	All patients (*n*= 613)	Survivors (*n*= 370)	Non-Survivors (*n*= 243)	*P*
General occurrences				
Need of MV (days)	9 (4 – 17)	8 (2 – 14)	10 (5 – 20)	<0.001
Intravenous contrast	65 (10.6%)	37 (10.0%)	28 (11.5%)	0.642
Blood transfusion	257 (41.9%)	134 (36.2%)	123 (50.6%)	0.001
Surgery	213 (34.7%)	122 (33%)	91 (37.4%)	0.293
Infection	291 (47.5%)	167 (45.1%)	124 (51%)	0.178
Sepsis	163 (26.6%)	61 (16.5%)	102 (42%)	<0.001
Biochemical imbalances				
Hyponatremia	449 (73.2%)	268 (72.4%)	181 (74.5%)	0.639
Hypernatremia	219 (35.7%)	109 (29.5%)	110 (45.3%)	<0.001
Hypokalemia	398 (64.9%)	240 (64.9%)	158 (65%)	0.963
Hyperkalemia	275 (44.9%)	103 (27.8%)	172 (70.8%)	<0.001
Hypoglycemia	104 (17%)	50 (13.5%)	54 (22.2%)	0.007
Hyperglycemia	487 (79.4%)	272 (73.5%)	215 (88.5%)	<0.001
Hypochloremia	159 (25.9%)	88 (23.8%)	71 (29.2%)	0.159
Hyperchloremia	484 (79%)	293 (79.2%)	191 (78.6%)	0.941
Metabolic acidosis	469 (76.5%)	252 (68.1%)	217 (89.3%)	<0.001
Need of drugs				
Vasoactive drugs	516 (84.2%)	280 (75.7%)	236 (97.1%)	<0.001
Diuretics	505 (82.4%)	280 (75.7%)	225 (92.6%)	<0.001
NSAIDs	314 (51.2%)	216 (58.4%)	98 (40.3%)	<0.001
Vancomycin	76 (12.4%)	42 (11.4%)	34 (14%)	0.398
Amphotericin B	28 (4.6%)	12 (3.2%)	16 (6.6%)	0.082
Polymycin B	73 (11.9%)	34 (9.2%)	39 (16%)	0.015
Aminoglycosides	167 (27.2%)	95 (25.7%)	72 (27.6%)	0.326
Fluid balance				
Oliguria (days)	0 (0 – 1)	0 (0)	1 (1 – 3)	<0.001
Negative fluid balance (days)	4 (1 – 7)	5 (2 – 8)	2 (0 – 5)	<0.001
Positive fluid balance (days)	8 (4 – 15)	7 (3 – 14)	8 (5 – 18)	0.001
Accumulated fluid balance (liters)	+7.3	+3.8	+13.5	<0.001
	(+1.5 – +18.3)	(-433 – +12.37)	(+6.6 – +28)	
Organ dysfunction				
Hematologic	243 (39.6%)	107 (28.9%)	136 (56%)	<0.001
Cardiovascular	255 (41.6%)	28 (7.6%)	227 (93.4%)	<0.001
Hepatic	254 (41.4%)	115 (31.1%)	139 (57.2%)	<0.001
Respiratory	293 (47.8%)	59 (15.9%)	234 (96.3%)	<0.001
Multiple	313 (51.1%)	81 (21.9%)	232 (95.5%)	<0.001

MV: mechanical ventilation; ICU: intensive care unit; NSAIDs: nonsteroidal anti-inflammatory drugs.

The non-survivor group had AKI diagnosis carried out mainly by KDIGO criteria (*n=* 134, 55.2% *vs.* survivor group: *n*= 63, 17%; *p*-value <0.001), more sepsis-associated AKI (*n*= 69, 28.4% *vs.* survivor group: *n*= 59, 15.9%; *p*-value <0.001), RRT need (*n*= 47, 19.3% *vs*. survivor group: *n*= 29, 7.8%; *p*-value <0.001), and higher frequency of non-recovery of renal function (*n*= 221, 90.9% *vs.* survivor group: *n*= 54, 14.6%; *p*-value <0.001) (Table 3).

**Table 3:** Data of AKI diagnosis.

Variables	All patients (*n*= 631)	Survivors (*n*= 370)	Non-Survivors (*n*= 243)	*p*
KDIGO criteria for diagnosis				**<0.001**
Serum creatinine	343 (56%)	261 (70.5%)	82 (33.7%)	
Urine output	73 (11.9%)	46 (12.5%)	27 (11.1%)	
Both	197 (32.1%)	63 (17%)	134 (55.2%)	
Associated etiology				**<0.001**
Sepsis	128 (20.9%)	59 (15.9%)	69 (28.4%)	
Septic shock	58 (9.5%)	27 (7.3%)	31 (12.8%)	
Multiple organ dysfunction	71 (11.6%)	5 (1.4%)	66 (27.2%)	
Nephrotoxic	100 (16.3%)	78 (21.1%)	22 (9.1%)	
Major surgery	134 (21.9%)	120 (32.4%)	14 (5.8%)	
Hypovolemia	20 (3.3%)	13 (3.5%)	7 (2.9%)	
Rhabdomyolysis	37 (6%)	30 (8.1%)	7 (2.9%)	
RRT need	76 (12.4%)	29 (7.8%)	47 (19.3%)	**<0.001**
Recovery of renal function				**<0.001**
Total	264 (43.1%)	252 (68.1%)	12 (4.9%)	
Partial	74 (12.1%)	64 (17.3%)	10 (4.1%)	
Non-recovery	275 (44.9%)	54 (14.6%)	221 (90.9%)	

AKI: acute kidney injury; KDIGO: Kidney Disease Improving Global Outcomes; RRT: renal replacement therapy.

The multivariate logistic regression results showed that the independent factors associated with mortality were the need for vasoactive drugs during the ICU stay (odds ratio (OR) = 11.49 [2.46-53.70]; *p*-value 0.002), diagnosis of major surgery-associated AKI (OR= 16.22 [3.49-75.38]; *p*-value <0.001), and non-recovery of renal function (OR= 92.7 [38.43-223.62]; *p*-value = <0.001) (Table 4).

**Table 4:** Mortality risk factors by multivariate logistic regression.

Variables	OR	CI (95%)	p
Time with oliguria (days)	0.93	0.93 – 1.03	0.517
AKI diagnosis by UO	1.25	0.44 – 3.52	0.672
Aki diagnosis by UO and SCr	2.27	1.10 – 4.72	0.027
Gender (male)	0.75	0.39 – 1.44	0.379
Nosocomial infection	0.95	0.45 – 2.01	0.892
Oliguria	2.14	0.62 – 7.37	0.228
Hypernatremia	1.53	0.76 – 3.07	0.230
Hyperkalemia	3.6	1.78 – 7.30	0.000
Hypoglycemia	2.11	0.90 – 4.96	0.087
Metabolic acidosis	2.8	1.13 – 6.92	0.026
Vasoactive drug	11.49	2.46 – 53.7	0.002
NSAIDs	0.83	0.43 – 1.60	0.573
KDIGO Stage 3	3.21	1.26 – 8.24	0.015
Hepatic dysfunction	1.76	0.88 – 3.51	0.108
Partial renal recovery	2.77	0.89 – 8.59	0.078
Non-recovery	92.7	38.43 – 223.62	<0.001
RRT need	0.45	0.17 – 1.22	0.116
Nephrotoxic	1.44	0.48 – 4.37	0.516
Major surgery	16.22	3.49 – 75.38	0.000
Shock	0.29	0.10 – 0.87	0.028
Multiple dysfunction	0.36	0.12 – 1.08	0.069
Rhabdomyolysis	5.2	0.87 – 31.17	0.071

NSAIDs: non-steroids anti-inflammatory drugs; UO: urine output. SCr: serum creatinine; KDIGO: Kidney Disease: Improving Global Outcomes.

**Figure 2:**
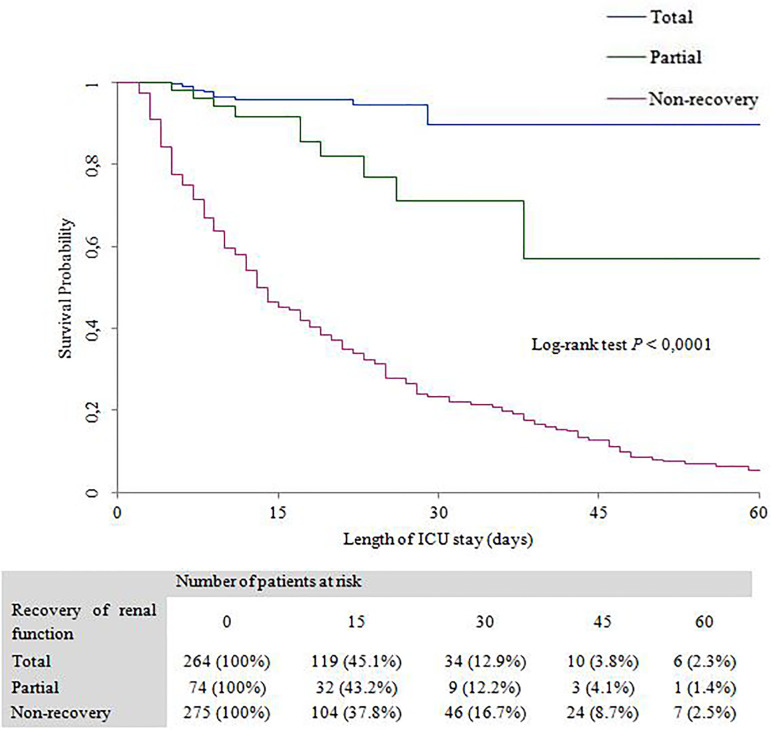
Kaplan-Meier curves stratified by recovery of renal function post-AKI in ICU.

Figure 2 shows the patient’s survival rate by Kaplan-Meier curves according to the recovery of renal function after AKI in ICU.

## Discussion

In Brazil, epidemiologic studies about AKI are still incipient, but the event is frequent among critically ill patients in the ICUs and the negative outcome rate is high.^9^^-^ 10 ^,^ 15 ^-^ 17. We carried out a comprehensive analysis of 613 AKI patients diagnosed by KDIGO^1^ criteria, with a focus on mortality and its risk factors.

Data were analyzed from patients with general clinical-epidemiological features, who stayed in one mixed ICU from a university-affiliated hospital, the majority admitted to the ICU with a surgical diagnosis (58.6%) (Table 1). In this study, the global mortality rate was 39.6% and increased progressively according to the severity of the event (Fig. 1). In earlier research conducted in the same region as our study, the AKI mortality rate was 52% in general, and 84.2% among patients with the need for RRT. In that study, the APACHE II score median was 21 points and the median length of ICU stay was 8.5 days.^10^

Other researchers carried out a prospective study of 498 patients admitted to the general ICU of a university-affiliated hospital located in the Brazilian Southeast, with clinical-epidemiological features similar to the patients included in our study. The mortality among AKI patients was 62.1%.^17^

During the ICU stay, the non-survivor group had greater accumulated fluid balance (in liters) (+3.9 [-0.433 - +12.37] *v*s. +13.5 [+6.6 - +28]; *p-*value: <0.001) (Table 2), as well as higher sepsis-associated AKI (42% *vs* 16.5%; *p-*value: <0.001) (Table 3).

Fluid overload is not only associated with AKI but can also be its result and it is pointed out as a potential prognostic biomarker of AKI, considering that its occurrence precedes the increase in creatinine and the decrease in diuresis.^18^^-^ 21 Besides, many patients may have an underestimated creatinine level due to hemodilution caused by fluid overload, which restricts the identification of AKI through the application of current diagnostic criteria.^18^ In an attempt to minimize this bias, Macedo et al.^19^ and, more recently, Thongprayoon et al.^20^ proposed the application of formulas to calculate the creatinine level adjusted for the positive fluid balance. Another way of defining fluid overload is to assess the increase in the percentage of the patient’s baseline bodyweight.^21^

Besides, it is known that there is an association between fluid overload and greater negative outcomes in AKI patients, including death.^18^^-^ 22 Considering that septic patients can benefit from volume expansion in the early hours, it is emphasized that volume replacement should occur until the intravascular volume is restored, with minimum fluid administration.^22^

Sepsis is the main AKI cause,^5^ substantially increases the risk of death in the critically ill patients, both in adult and pediatric populations,^23^ and contributes to the progression of chronic kidney disease after AKI.^24^ We did not include sepsis occurrence and sepsis-associated AKI in the multivariate models of mortality due to multicollinearity.

In Table 4, we show that non-recovery of renal function at ICU discharge was significantly associated with death among AKI patients. The pathophysiology of renal recovery after an AKI episode involves several mechanisms, including cell cycle arrest, infiltration of inflammatory cells, stimulation of fibrocytes and myofibroblasts, as well as secretion of inflammatory cytokines.^14^

The non-recovery of renal function after AKI has been related to several negative outcomes, and the severity and duration of the AKI can influence the recovery degree.^14^^,^ 25 In a group of Brazilian patients with dialysis AKI, the non-recovery of renal function was associated with a higher mortality rate.^26^^)^ In another Brazilian study, after 30 days of follow-up, AKI patients who did not require dialysis showed greater recovery of renal function.^27^^)^ In a population-based study carried out in Canada, non-recovery of renal function was associated with death and other adverse renal events in the long term, including end-stage renal disease (ESRD).^28^

Age, comorbidities, and some genetic aspects are among the risk factors for non-recovery of renal function after AKI.^14^^,^ 25 The impact of the event on distant organs also plays an important role, as AKI is responsible for lung (edema and acute injury), cardiac (arrhythmia, congestive heart failure, and ischemic heart disease), brain (uremic encephalopathy, dementia, and stroke), hepatic (altered hepatic metabolism), intestinal (uremic toxin accumulation and altered gut microbiota), and immune system (systemic inflammation) dysfunctions, and the non-recovery of the renal function may stress the hemodynamic, humoral, and immunologic changes.^26^

In this sense, delays in the recognition and treatment of clinical complications (fluid overload, inflammation/infection, acidosis, electrolyte abnormalities, and other local and distant organ complications) in combination with ineffective care (improper antimicrobial therapy, as well as improper metabolic and nutritional support) are determinants of the non-recovery of renal function and, consequently, explain the role of this condition as a strong independent risk factor associated with mortality in AKI patients.^27^

AKI occurrence after major surgery also showed an independent association with mortality among patients. As in other regions of the world, postoperative AKI in Brazil is described mostly after cardiac surgery, with high incidence and mortality rates.^28^^-^ 30 Considering that our institution is a reference in trauma, empirically, we know that postoperative AKI is mainly associated with neurologic, abdominal, and orthopedic surgeries.

The use of vasoactive drugs during ICU stay (OR: 11.49; CI: 2.46-53.7), occurrence of hyperkalemia (OR: 3.6; CI: 1.78-7.30), and metabolic acidosis (OR: 2.8; CI: 1.13-6.92) were modifiable factors significantly associated with mortality in the patients studied. KDIGO stage 3 (OR: 3.21; CI: 1.26-8.24) and AKI diagnosis by both KDIGO criteria (OR: 2.27; CI: 1.10-4.72) (see Table 4) were also associated to death (Table 4). These results are similar to the data observed in other Brazilian studies, as well as in large international cohorts.^5^^-^ 10 ^,^ 15 ^-^ 17 ^,^ 31 ^-^ 32

Despite the robust and growing number of studies carried out around the world, the Brazilian literature on AKI in the ICU is still incipient and many aspects need further study in our country. We conducted a comprehensive epidemiologic study to investigate the factors associated with death in AKI patients in the ICU of a Brazilian center. Nevertheless, we recognized that our study had some limitations, including the retrospective design and data from a single center. Unfortunately, the lack of data prevented us from performing subgroup analyses and limited our ability to further reduce confounding biases. We neither assessed the AKI duration nor factors associated with renal recovery after AKI. Thus, in future studies, these aspects should be addressed.

## Conclusion

The mortality rate in the AKI patients in this study was similar to other studies and the factors significantly associated with death were verified. Further research is necessary to confirm the findings through multicenter studies with a prospective design. For the appropriate management of AKI in the ICU, early recognition of determinant factors of incidence and mortality is essential. Also, it is important to eliminate risk factors for AKI in patients who are at risk for and/or have been diagnosed with AKI.
